# Enhanced Recovery after Vascular Surgery

**DOI:** 10.3389/fmed.2018.00002

**Published:** 2018-01-19

**Authors:** Milena D. Stojanovic, Danica Z. Markovic, Anita Z. Vukovic, Vesna D. Dinic, Aleksandar N. Nikolic, Tijana G. Maricic, Radmilo J. Janković

**Affiliations:** ^1^Center for Anesthesiology, Reanimatology and Intensive Care, Clinical Center Nis, Nis, Serbia; ^2^School of Medicine, University of Nis, Nis, Serbia

**Keywords:** vascular surgery, recovery, postoperative management, intensive care, preoperative care

## Abstract

The beginnings of the enhanced recovery after surgery (ERAS) program were first developed for patients in colorectal surgery, and after it was established as the standard of care in this surgical field, it began to be applied in many others surgical areas. This is multimodal, evidence-based approach program and includes simultaneous optimization of preoperative status of patients, adequate selection of surgical procedure and postoperative management. The aim of this program is to reduce complications, the length of hospital stay and to improve the patients outcome. Over the past decades, special attention was directed to the postoperative management in vascular surgery, especially after major vascular surgery because of the great risk of multiorgan failure, such as: respiratory failure, myocardial infarction, hemodynamic instability, coagulopathy, renal failure, neurological disorders, and intra-abdominal complications. Although a lot of effort was put into it, there is no unique acceptable program for ERAS in this surgical field, and there is still a need to point out the factors responsible for postoperative outcomes of these patients. So far, it is known that special attention should be paid to already existing diseases, type and the duration of the surgical intervention, hemodynamic and fluid management, nutrition, pain management, and early mobilization of patients.

## Introduction

Enhanced recovery after surgery (ERAS) program or a “fast-track surgery” was first developed and successfully implemented in colorectal surgery (1), and was later reported in orthopedic (2, 3), cardiac (4), and vascular surgery (5, 6). The aims of this program are to improve the perioperative care of patients, avoid complications, accelerate recovery, shorten hospital stays and improve the patient’s prognosis. Introduced by Danish surgeon Dr. Henrick Kehlet, this multimodal evidence-based approach program starts in the preoperative period and extends to the patient’s release (1, 2).

It is possible to accelerate the patient’s recovery, reduce complications and the length of hospital stay by changing factors which are responsible for prolonged recovery (1, 7, 8). Previous studies have shown the safety and efficiency of ERAS program, and they emphasize that for early recovery after surgery following factors are important: patient education, short-acting anesthetics, pain management, fluid therapy, oral nutrition, and early mobilization (9, 10).

It is already proved in several studies that application of ERAS program reduces the length of hospital stay (11), decreases the surgical and non-surgical complications in postoperative period (12), and improves the outcome (13).

So far, the fields of interest in vascular surgery are ERAS in aortic (5) and carotid (6) surgery, but there are still not enough solid evidence to form a unique acceptable program.

## Preoperative Period

Preoperative assessment is as important as the intraoperative and postoperative period. It allows evaluation of risk and provides an opportunity to stabilize already existing disease and optimize organ dysfunction before surgery, as it is well known that postoperative organic dysfunction is undoubtfully associated with preoperative comorbidities. This period provides an opportunity for patient education, which refers to obtaining information about surgery itself, anticipated postoperative course, analgesia, and discharge. It is thought that in this way anxiety, the need for analgesics and the length of hospital stay can be reduced (1). The recommendations for ERAS in vascular surgery (including preoperative, intraoperative, and postoperative period) are shown in Table 1.

**Table 1:** Recommendations for enhanced recovery program in vascular surgery.

Respiratory
Preoperative antibiotics use
Early extubation
Protective modes of ventilation
Prevention of ventilator-associated pneumonia
High-flow oxygen therapy or intermittent nasal continuous positive airway pressure after extubation
Breathing exercises

**Cardiovascular**
Monitoring for signs of myocardial ischemia
Goal directed fluid therapy
Maintaining the MAP above 80–90 mmHg

**Renal**
Monitoring of the amount of urine and creatinine clearance
Maintaining the normovolemia and electrolyte balance
Use diuretics with caution
Avoid fluid overload and dopamine

**Nutrition**
Without preoperative mechanical bowel preparation
Maintenance the glucose level < 215 mg/dl
Oral nutrition within the first 24–48 h after operation
Use prokinetics (metoclopramid and erithromycin)
Avoidance of nasogastric drainage or early removal
Monitoring of IAP

**Pain management**
PCEA 48 h before and 48 h after intervention
Avoid systemic opioid use

**Other**
Stabilize already existing disease and optimize organ dysfunction before surgery
Oral carbohydrate drinks before surgery
Minimize the time for surgical intervention
Patient education
Consider thromboprophylaxis
Avoid hypothermia
Early mobilization

Special attention should be paid to premedication with an aim to reduce the stress response to surgery. Beta-blockers reduce the catecholamine level and thus reduce the perioperative and postoperative cardiovascular complications. Since surgical intervention is a stress that leads to activation of the sympathetic nervous system and catabolism, these medications with their anti-catabolic effect could have a positive impact on postoperative course. It is considered that alpha-2 adrenergic agonists, such as clonidine and dexmedetomidin, reduce myocardial ischemia, intraoperative blood loss, and postoperative nausea and vomiting (1).

Preoperative overnight fasting can lead to dehydration, and its avoidance reduces the risk of postoperative pain and nausea. It has even been proved that clear carbohydrate fluids given during this period can reduce the postoperative anxiety and endocrine response (1, 14).

## Intraoperative Period

One of the major interventions in vascular surgery is an aortic surgery. Today available surgical techniques are endovascular and open repair. Study form Hertzer et al. showed that after elective open infrarenal aortic repair median length of stay in intensive care unit (ICU) is about 3 days and mortality rates are between 1.2 and 10.5% (15). Postoperative complications such as aneurysm rupture, thrombosis and graft migration are same after both surgical techniques, while endovascular repair has advantages in terms of length of intervention, blood loss, shorter length of mechanical ventilation, less malnutrition, and shorter hospital stay (16–18).

Special attention should be directed to intraoperative heat loss in order to avoid hypothermia and patient warming system should be strongly considered (16).

## Postoperative Period

A postoperative period in vascular surgery plays a pivotal role in the patient’s recovery. The quality of postoperative care is essential for the successful recovery. Following the interventions in vascular surgery the most significant are considered to be: optimization of microcirculation after ischemic–reperfusion syndrome and inflammatory reaction caused by the operation itself, adequate fluid resuscitation, maintenance of satisfactory aerobic metabolism, stable blood glucose levels and adequate oxygen delivery, flow directed hemodynamic support, and use of vasodilators and vasoconstrictors with the goal of achieving adequate blood flow (19).

The spectrum of complications depends on the disease itself, its urgency and the surgical procedure (16). Some of these complications can appear in the early postoperative phase, but some of them can be manifested in the late postoperative period.

Immediately after operation, Crimi and Hill in their study point out the importance of resolving hemodynamic, pulmonary, renal, neurological, hematological, and gastrointestinal complications (20). The disorders of these organ systems are the result of ischemic–reperfusion injury.

Managing in the first postoperative day, mainly depends on the type of surgical intervention, the length of operation and the condition of the patient. During this period, it is important to be cautious about.

### Hemodynamic Stability

Hemodynamic instability often occurs after major and complicated surgery. It is preferable to avoid hypertension, yet allowing adequate perfusion of vital organs. For this purpose, a combination of nitrloglycerine (NTG) and labetalol with some inodilators such as dobutamine is mostly recommended (19). The patients are often vasoplegic and hyperdynamic and require vasoconstrictors in the form of noradrenaline or vasopressin. This requires adequate invasive monitoring or the placement of pulmonary catheter. Oxygen delivery markers such as SvO_2_ and serum lactate levels should also be monitored. Transesophageal ultrasound should be available, especially during the periods of hemodynamic instability (19).

### Respiratory Failure

Respiratory failure, most commonly caused by infection and pulmonary pathology, is still the most common complication after surgery of thoracoabdominal aorta and occurs in about 30% of patients (20). Respiratory function is often most stable shortly after surgery and patients can usually be extubated within 6–12 h or even earlier. Only in rare cases of transfusion-related acute lung injury, or pulmonary infection exacerbation in high-risk patients, gas exchange remains compromised and requires prolonged ventilatory support. Additionally, major surgery and preexisted kidney disease can lead to significant pulmonary complications (21). If there is a risk of volotrauma, low tidal volumes can be used. Hypercapnia and respiratory acidosis are logical consequence of this ventilation mode, but if the patient does not have cerebral edema, this permissive hypercapnia is generally well tolerated with pH > 7.2 (22). If there is a need for lung recruitment and after separating from the ventilator, it is preferable to use high-flow oxygen therapy or intermittent nasal continuous positive airway pressure (19).

Factors such as: extreme age, comorbidities, extensive surgery, prolonged use of muscle relaxants and sedatives, preexisting pulmonary disease, postoperative hypothermia and fluid overload can lead to V/Q mismatch, hypoxia, and respiratory failure. Prevention of respiratory complications should start in the preoperative period using antibiotics and breathing exercises (22).

### Myocardial Ischemia

Study of Landesberg et al. showed that transient myocardial ischemia developed among 21% of patients and myocardial infarction in 6.5% of cases (23). Combination of two precordial leads are more than 95% sensitive than troponin level for postoperative ischemia monitoring. If myocardial ischemic event occurs, supplemental oxygen, beta-blockers, afterload reduction agents, anticoagulants and antiplatelets should be administered. Percutaneous coronary intervention is also recommended, while postoperative fibrinolysis is a relative contraindication (22). If ischemic events are associated with signs of myocardial impairments, manifested by an increased need for inotropic use, reduced cardiac output (CO), cardiac arrhythmias or disorders of wall motility, urgent angiography can be indicated (19).

### Bleeding and Coagulopathy

A typical consequence of aortic surgery is coagulopathy with subsequent bleeding. Early stabilization of coagulopathy is important in prevention of further complications. Special point-of-care laboratory tests such as thromboelastography or activated clotting time allow the treatment of such disorders (19). Preoperative administration of antifibrinolytics can be continued in the short postoperative period.

Liberal blood transfusions for treatment of massive bleeding are no longer in use because of high 30 days’ adverse events among these patients. It is recommended that if hemoglobin is above 9 g/dl, transfusion should be avoided. When there is a need for correction of clotting factors deficiency, the dose of fresh frozen plasma should be 10–15 ml/kg of body weight, with a maximum dose of 30 ml/kg, and most important is that fresh frozen plasma should not be used as a volume expander (22).

### Temperature Management

Immediately after surgery patients are often hypothermic, especially after long interventions. Maintaining an adequate body temperature in postoperative period is important for adequate oxygen supply, functioning of the coagulation system, hemodynamic stability, and neurocognitive integrity (19).

### Neurologic Disorders

Typical complications following aortic surgery are stroke, spinal cord ischemia or generalized cognitive dysfunctions presented as delirium or confusion/agitation (19, 20). Study from Beydon et al. showed that lorazepam in premedication was associated with prolonged extubation time and a lower rate of cognitive recovery (24). But Scavee et al. showed that fear and anxiety increase the incidence of postoperative complications and that use of anxiolytics can be desirable (25).

### Deep Vein Thrombosis (DVT)

The risk of DVT after open abdominal aortic aneurysm repair (AAA) is 2–33% without chemoprophylaxis, and 1–10.2% in patients who received postoperative enoxaparin (26). Previous studies, which had some limitations, have shown that because of low incidence of postoperative DVT or high risk of bleeding, there is no need for postoperative thromboprophylaxis (26).

Scarborough et al. in their study (6,000 patients) showed that the incidence of DVT after AAA is only 2.4%, but the limitation in this study was that they did not know which patient received thromboprophylaxis. They emphasize that the risk factors for DVT after open surgery are: length of surgery more than 5 h, obesity and ruptured aneurysmal disease (27).

For the time being, there are no unique guidelines for thromboprophylaxis. Although many studies in this field were conducted, they included small number of patients, so their conclusions cannot be considered as relevant.

Based on available data, the majority of vascular surgeons consider that the use of thromboprophylaxis should become a routine for patients undergoing AAA repair (27).

Special attention should be paid to patients with carotid surgery, especially those who develop some neurological deficit. Due to uncontrolled hypertension, cerebral hyperperfusion syndrome can develop, and this should be controlled with IV labetalol. Hypotension should be treated with volume infusion and phenylephrine (22).

In the late postoperative period the focus should be put on acute kidney injury (AKI) and intra-abdominal complications.

### Acute Kidney Injury

The most common predictor factors for AKI are physiological reserve as well as the severity of atherosclerotic disease (28, 29). The incidence of postoperative AKI requiring dialysis ranges from 5 to 15%, and its occurrence is associated with a worse prognosis (30). Monitoring the amount of urine as well as creatinine clearance in postoperative period is crucial. Diuretics and dopamine can promote urine output, but integrity or functional reserve of glomerular function may be compromised (31). Patients with intra-abdominal pressure >25 mmHg are at risk for compartment syndrome development, which may cause AKI. In this group of patients it is important to maintain higher values of blood pressure and better oxygen delivery (22).

### Intra-Abdominal Complications

Some of these complications are rare, such as mesenteric ischemia (2–5%), but the mortality rate is high (55–60%) (32). Cross-clamping the aorta may cause mesenteric ischemia with consequent reperfusion injury, translocation of the bacteria and systemic inflammatory response. After cardiopulmonary bypass, low CO can cause gut ischemia (where local vasodilatators, such as nitric oxide and papaverine, can be helpful) and colonic infarction with high mortality rate (89%) (33).

## Guidelines for Management in Postoperative Period

### Hemodynamic Management

Hypoxia and hypertension in postoperative period can be harmful for patients. Hypertension is associated with increased incidence of strokes or aorta dissection. On the other hand, hypotension is associated with graft thrombosis and multiorgan failure, therefore, maintenance of adequate oxygen supply is crucial for kidneys, CNS, and spinal cord (22). The recommendations are to keep mean arterial pressure above 80–90 mmHg, as well as maintenance the systolic blood pressure above 130 mmHg. Achieving these values of blood pressure is not always easy in practice and often requires the use of vasodilatatotors—NTG and labetalol, vasoconstrictors—noradrenaline and vasopressin, and even inotropes such as dobutamin. Even with this support, fluid replacement can be a real challenge (19). Recent recommendations are based not only on the satisfactory pressure maintenance but also on tissue perfusion which is equally important (34). Among various types of arrhythmias, bradiarrythmias should not be treated if they are not associated with hemodynamic instability (35, 36).

Hemodynamic management is now directed not only toward the maintenance of satisfactory tissue perfusion but also toward flow directed approach, with clinical assessment of urine output and oxygen extraction index (19, 37). Recently, it has been showed that diuretics can improve venous drainage from the microcirculation, increase the oxygen extraction at cellular level and improve mitochondrial function (37).

### Fluid Management

Many patients after operation do not have the ability to excrete fluids and sodium; on the other side, many of them are hypovolemic in this period (38). Crystalloid solutions like Ringer-lactate should be favourized during this period. Normal saline should be avoided due to hyperchloremic metabolic acidosis, which can reduce tissue perfusion and worsen the final outcome (19). In general, colloids are better in raising the blood pressure and improving the tissue perfusion, but they should be used with great caution due to renal toxicity and unfavorable effects on coagulation system (19). With limited evidence, it is advised to avoid them in patients with an already impaired kidney function (39).

### Nutrition

Postoperative insulin therapy with the goal of maintenance the glucose level < 215 mg/dl (11.9 mmol/l) is mandatory for diabetic and non-diabetic patients (40). The relationship between high glucose levels and worse outcomes is a well-known concept. Study from Krinsley et al. showed that the mortality is twice as high when glucose level is above 140–150 mg/dl (41). An increase of glucose level for every 40 mg/dl carries a 30% higher risk of infection, graft failure, and longer ICU stay (42). Achieving these values is significant for wound healing, integrity of gastrointestinal system, and inflammatory response reduction (43).

Malnutrition in preoperative period is associated with muscle weakness, fatigue, immunological dysfunction, and slower wound healing. Some studies have proven that adequate nutrition even in the preoperative period can improve surgical outcome (44, 45). Restoration of bowel motility plays a very important role in the ERAS program since the beginning of food intake depends on it. Enteral feeding has fewer metabolic complications than parenteral nutrition (22), and it should be started as soon as possible in the postoperative period (6–8 h) (46). On the contrary, Ksienski et al. showed in their study that early oral nutrition should start within the first 24–48 h while gastric emptying occurs within 18 h after elective aortic aneurysm repair (47). If there is a postoperative disturbance in bowel motility, first-line choice therapy for prokinetic medications are metoclopramid and erythromycin (18).

The use of nasogastric drainage (ND) is not recommended and can even be harmful. It has been shown that in patients without ND bowel function returns earlier and that they have fewer pulmonary complications (18, 48).

### Pain Management

Pain management in postoperative period is very important and one Cohrane review showed that use of epidural analgesia has fewer cardiovascular and renal complications compared with systemic opioid use with no difference in mortality (20). Patient-controlled epidural analgesia (PCEA) with local anesthetics provides better analgesic effect than patient-controlled analgesia (PCA) with intraveonous opioids, but there is no statistical difference in hospital stay and its outcome when these two techniques are compared (49). Additionally, based on available evidences for ERAS protocol, the PCA epidural analgesia is highly recommended (50). PCEA should begin 48 h before intervention and continue 48 h after operation (51). Criteria for discharge are: hemodynamic stability, urine output >0.5 ml/kg/h, adequate analgesia to make patients active, ability to consume solid fluid, and no indications for further surgical interventions (23, 49).

## Conclusion

Although ERAS program was most studied and applied in colorectal surgery, recent studies suggest that it can be successfully applied in vascular surgery, too. The most important is establishing the guidelines and form a team whose members (surgeons, anesthesiologists, nurses, rehabilitation members, and nutritionists) should be familiar with ERAS program and must be motivated to carry out the program.

Since the field of vascular surgery is too broad, one cannot even expect that the conceptualization of just one program is sufficient for all areas in vascular surgery. For the moment, some acceptable guidelines for the postoperative management already exist, therefore, it is necessary to direct the attention to the preoperative status of a patient and improvements in surgical techniques.

## Author Contributions

RJ conceived of the presented idea. MS, AV, VD, and DM wrote the beginning version of the manuscript. MS, AV, RJ, VD, DM, AN, and TG contributed to the design of the research and the writing of the final version of the manuscript. RJ supervised the manuscript.

## Conflict of Interest Statement

The authors declare that the research was conducted in the absence of any commercial or financial relationships that could be construed as a potential conflict of interest. The handling editor declared a shared affiliation, though no other collaboration, with one of the authors RJ.

## Abbreviations

ERAS, enhanced recovery after surgery; ICU, intensive care unit; DVT, deep vein thrombosis; AAA, abdominal aortic aneurysm; AKI, acute kidney injury; NTG, nitrloglycerine; MAP, mean arterial pressure; ND, nasogastric drainage; PCA, patient-controlled analgesia; PCEA, patient-controlled epidural analgesia; CO, cardiac output.
